# Generation of Dipeptidyl Peptidase-IV-Inhibiting Peptides from *β*-Lactoglobulin Secreted by *Lactococcus lactis*

**DOI:** 10.1155/2014/393598

**Published:** 2014-08-03

**Authors:** Suguru Shigemori, Kazushi Oshiro, Pengfei Wang, Yoshinari Yamamoto, Yeqin Wang, Takashi Sato, Yutaka Uyeno, Takeshi Shimosato

**Affiliations:** ^1^Interdisciplinary Graduate School of Science and Technology, Shinshu University, 8304 Minamiminowa, Kamiina, Nagano 399-4598, Japan; ^2^Japan Society for the Promotion of Science (JSPS), Japan; ^3^Graduate School of Agriculture, Shinshu University, 8304 Minamiminowa, Kamiina, Nagano 399-4598, Japan; ^4^Department of Internal Medicine and Clinical Immunology, Graduate School of Medicine, Yokohama City University, 3-9 Fukuura Kanagawa, Yokohama 236-0004, Japan; ^5^Frontier Agriscience and Technology Center (FAST), Shinshu University, 8304 Minamiminowa, Kamiina, Nagano 399-4598, Japan; ^6^Graduate School of Agriculture, Department of Interdisciplinary Genome Sciences and Cell Metabolism, Institute for Biomedical Sciences, Interdisciplinary Cluster for Cutting Edge Research (ICCER), Shinshu University, 8304 Minamiminowa, Kamiina, Nagano 399-4598, Japan

## Abstract

Previous studies showed that hydrolysates of *β*-lactoglobulin (BLG) prepared using gastrointestinal proteases strongly inhibit dipeptidyl peptidase-IV (DPP-IV) activity *in vitro*. In this study, we developed a BLG-secreting *Lactococcus lactis* strain as a delivery vehicle and *in situ* expression system. Interestingly, trypsin-digested recombinant BLG from *L. lactis* inhibited DPP-IV activity, suggesting that BLG-secreting *L. lactis* may be useful in the treatment of type 2 diabetes mellitus.

## 1. Introduction

*β*-Lactoglobulin (BLG) is the major whey protein in the milk of cows and other ruminants, as well as some nonruminants, but it is not contained in human breast milk [1]. BLG is widely recognized as a high-functional protein [2]. Previous studies have consistently demonstrated that hydrolysates of BLG prepared using gastrointestinal proteases such as trypsin or pepsin have an inhibitory effect on dipeptidyl peptidase-IV (DPP-IV, also known as CD26) activity [3–8]. DPP-IV is a serine protease that is ubiquitously expressed* in vivo*, and its endogenous physiological substrates are incretins [9]. The incretins, primarily glucose-dependent insulinotropic polypeptide and glucagon-like peptide-1, are gastrointestinal hormones released from intestinal L and K cells, respectively. Incretins drive insulin secretion in pancreatic *β* cells and suppress pancreatic glucagon production [10, 11]. Thus, DPP-IV inhibitors, such as vildagliptin, saxagliptin, sitagliptin, alogliptin, and linagliptin, are used in the management of type 2 diabetes mellitus (T2D).

We hypothesized that intestinal mucosa-targeted oral delivery of BLG might be a useful strategy for managing T2D. Generally recognized as safe (i.e., GRAS), genetically modified lactic acid bacteria (LAB) have recently emerged as vehicles for the efficient delivery of therapeutic proteins and as stable producers of these proteins* in situ *[12]. In this study, we engineered a* Lactococcus (L.) lactis* strain to secrete BLG and validated the DPP-IV-inhibiting activity of trypsin-digested recombinant BLG (rBLG).

## 2. Materials and Methods

### 2.1. Bacterial Strains and Growth Conditions

*L. lactis* NZ9000 (NZ9000; MoBiTec, Goettingen, Germany), derived from* L. lactis* subsp.* cremoris* MG1363 (MG1363), which harbors the regulatory genes* nisR* and* nisK* integrated into the* pepN* gene, was used as a host strain. NZ9000 was grown at 30°C without shaking in M17 broth (OXOID, Hampshire, UK) supplemented with 0.5% glucose (GM17). rNZ9000 strains were grown in GM17 with 20 *μ*g/mL of chloramphenicol.

### 2.2. Construction of rNZ9000 Strains

**Figure 1:**
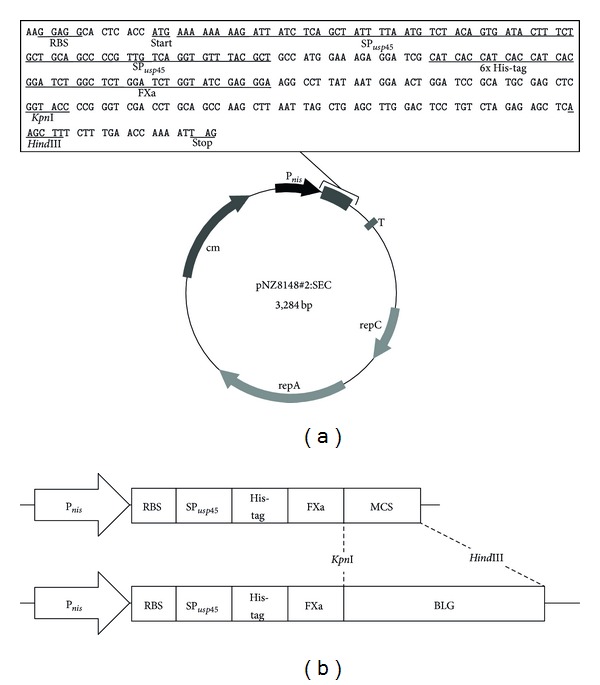
Maps of vectors used in this study. (a) Schematic representation of the secretion vector, pNZ8148#2:SEC. The DNA sequence between the RBS and the stop codon is presented in the rectangle. (b) The codon-optimized BLG gene (537 bp) was cloned into pNZ8148#2:SEC (above) between the* Kpn*I and* Hind*III restriction enzyme recognition sites, and the resulting plasmid was designated pNZ8148#2:SEC-BLG (below). *P*_nis_: nisin-inducible promoter; RBS: ribosome binding site; SP_*usp*45_: sequence of the signal peptide of the* usp45* gene product; His-tag: 6x histidine-tag; FXa: factor Xa recognition site; MCS: multiple cloning site; T: terminator; rep: replication gene; cm: chloramphenicol acetyltransferase gene; BLG: *β*-lactoglobulin.

To create the secretion vector for* L. lactis*, the sequence of* usp45*, the lactococcal signal peptide, was inserted into pNZ8148#2:CYT [13] between the nisin-inducible promoter (*P*_nis_) and 6x His-tag sequences; the resulting plasmid was designated pNZ8148#2:SEC (Figure 1(a)). The pNZ8148#2:CYT vector is a modified form of the* L. lactis* expression vector pNZ8148 (MoBiTec).

The gene encoding BLG (accession number: EU883598) was synthesized and subcloned into pGEM-T easy optimized for MG1363 codon-usage by Eurofins Genomics (Tokyo, Japan). The sequence of the BLG gene was subsequently cloned between the* Kpn*I and* Hind*III restriction sites in pNZ8148#2:SEC, generating pNZ8148#2:SEC-BLG (Figure 1(b)).

The pNZ8148#2:SEC-BLG vector was introduced into NZ9000 by electroporation using a Gene Pulser Xcell electroporation system (Bio-Rad Laboratories Inc., CA, USA) following the manufacturer's instructions. The resulting recombinant strain was designated NZ9000:SEC-BLG. NZ9000 was also electroporated with the empty plasmid pNZ8148#2:SEC to generate an NZ9000 vector control strain (NZ9000:SEC-VC).

### 2.3. Small-Scale Expression of rBLG

A 1.9 mL aliquot of fresh medium was inoculated with 0.1 mL of an overnight culture and incubated at 30°C without shaking. When the turbidity of the culture reached an optical density at 600 nm (OD_600_) of 0.4 (1.5 to 2 h later), various concentrations of nisin were added and the culture was incubated for an additional 3 to 4 h. The OD_600_ of the culture was measured at the end of the incubation period.

Cells were isolated from the nisin-induced culture via centrifugation for 1 min at 8,000 ×g and 4°C. Protein extracts were prepared from the cells using previously described methods [14]. Proteins remaining in the supernatant were isolated and concentrated using trichloroacetic acid (TCA) precipitation. Briefly, 300 *μ*L of cold 100% (w/v) TCA solution was added to 1.5 mL of supernatant and the mixture was placed on ice for 3 h. The resulting precipitates were pelleted by centrifugation at 20,400 ×g and 4°C. To completely remove the TCA, the protein pellet was washed twice with ice-cold acetone. The resulting pellet was dried at 55°C and then dissolved in a small amount of TE buffer (10 mM Tris-HCl (pH 8.0), 1 mM EDTA). An equal volume of 2x sodium dodecyl sulfate-polyacrylamide gel electrophoresis (SDS-PAGE) sample buffer was added to each sample. The cell and supernatant fractions were boiled for 5 min or placed at room temperature overnight, respectively, and then subjected to SDS-PAGE (15% (v/v) polyacrylamide).

Electrophoresed proteins were transferred from the gel onto a Hybond-P polyvinylidene difluoride membrane (GE Healthcare, Buckinghamshire, UK). Western blotting was performed with primary antibody (Ab) against the 6x His-tag (1:1,000; BioLegend Inc., San Diego, CA, USA), followed by incubation with a horseradish peroxidase-conjugated secondary Ab (1:5,000; Sigma, St. Louis, MO, USA). The resulting blots were visualized using an ECL Western Blotting Analysis System (GE Healthcare). Signals were detected using a lumino image analyzer (ImageQuant LAS 4000 mini, GE Healthcare) and analyzed using ImageQuant TL software (GE Healthcare).

### 2.4. Purification of rBLG

Expression of rBLG in NZ9000:SEC-BLG was induced in a 4 L large-scale culture according to the above-mentioned methods. Following induction of rBLG expression, cells were harvested by centrifugation for 20 min at 3,000 ×g and 4°C. The cell pellet was washed twice with phosphate buffer (50 mM NaH_2_PO_4_, 300 mM NaCl, pH 8.0) and resuspended with 80 mL of phosphate buffer containing EDTA-free proteinase inhibitor cocktail (Roche Diagnostics, Indianapolis, IN, USA). To disrupt the cells, the bacterial suspension was homogenized with 0.2 mm glass beads for 3 min using a beads beater (Beads Crasher *μ*T-12, Taitec, Saitama, Japan) operating at 3,200 rpm. Soluble proteins released from the bacterial cells were collected by centrifugation for 15 min at 20,400 ×g and 4°C.

The rBLG was purified from the prepared lysate using a HisTrap HP column (1 mL, precharged with Ni^2+^; GE Healthcare) under native conditions. The phosphate buffer-equilibrated column was filled with the prepared lysate and then the flow-through was collected. Next, nonadsorbed proteins were eluted using wash buffer (phosphate buffer containing 20 mM imidazole). Adsorbed proteins were eluted with elution buffers (phosphate buffer containing 31, 63, 125, 250, or 500 mM imidazole). The prepurification crude lysate and the flow-through and eluted fractions were analyzed by Western blotting with an anti-6x His-tag Ab. Eluted fractions were dialyzed against Tris-buffered saline (TBS; 50 mM Tris-HCl (pH 8.0), 138 mM NaCl, and 2.7 mM KCl) and frozen at −80°C until use. The protein concentration of the dialyzed samples was measured with a BCA Protein Assay Kit (Thermo Scientific, Rockford, IL, USA) before the samples were frozen.

### 2.5. Trypsin Digestion of BLG

Commercial BLG (cBLG; Sigma) was dissolved in TBS at a concentration of 1 mg/mL. Purified TBS-dialyzed rBLG was diluted to 50 *μ*g/mL with TBS. Trypsin (from porcine pancreas, Wako Pure Chemical Industries Ltd., Osaka, Japan) was added at an enzyme to substrate ratio of 1 : 20 (w/w) and the mixture was incubated at 37°C for 24 h with gentle agitation. Digestion was stopped by heating the sample at 90°C for 5 min. The reaction mixture was kept frozen at −80°C until used in experiments.

### 2.6. Analysis of DPP-IV Inhibition

The DPP-IV inhibition assay was performed using a DPP-IV Drug Discovery Kit according to the manufacturer's instructions. Fluorescence resulting from degradation of substrate (H-Gly-Pro-AMC) by DPP-IV was detected using a Fluoroskan Ascent FL Microplate Fluorometer (Thermo Scientific) at excitation and emission wavelengths of 355 and 460 nm, respectively. Data are expressed as DPP-IV activity (%) remaining in test samples versus the control (no sample added).

### 2.7. cBLG-Immunized Mice

Pathogen-free female BALB/c mice (4 weeks of age, *n* = 3) were purchased from Japan SLC (Shizuoka, Japan) and housed under temperature- and light-controlled conditions. Mice were fed a standard diet (MF, Oriental Yeast Co., Ltd., Tokyo, Japan) and sterile water* ad libitum*. After a 1-week preliminary acclimatization period, mice were sensitized with 100 *μ*g of cBLG emulsified with 0.1 mL of complete Freund's adjuvant (Difco Laboratories, Detroit, MI, USA) and injected intraperitoneally. At 2 and 4 weeks after the first sensitization, mice were boosted with 100 *μ*g of cBLG emulsified with 0.1 mL of incomplete Freund's adjuvant (Difco Laboratories). All experimental procedures were conducted in accordance with the Regulations for Animal Experimentation of Shinshu University.

### 2.8. Immunoreactivity Assay

To investigate whether rBLG is immunoreactive, splenocytes were isolated from cBLG-immunized mice and the level of interleukin- (IL-) 13 mRNA expression was measured using real-time quantitative PCR (qPCR) following stimulation with purified rBLG. Splenocytes were prepared using standard methods [15, 16] and then cultured at a final concentration of 1 × 10^7^ cells/well (total volume of 2 mL per well). Splenocytes were stimulated with 50 or 100 *μ*g/mL of cBLG or purified rBLG. After 72 h of incubation, cells were harvested and IL-13 mRNA expression was assessed using qPCR as previously described [16]. Fluorescent qPCR was performed with SYBR Premix Ex Taq (TaKaRa Bio Inc., Tokyo, Japan) and IL-13-specific primers, with each reaction containing 5 ng of cDNA in a total volume of 25 *μ*L. The *β*-actin-specific and IL-13-specific primers were purchased from TaKaRa Bio Inc. The PCR cycling conditions were 10 s at 95°C followed by 45 cycles of 5 s at 95°C and 30 sec at 60°C. As a control, poly (A)^+^ RNA samples were used as templates to check for the presence of contaminating genomic DNA. Reaction sensitivity and amplification of contaminant products (e.g., extension of self-annealed primers) were evaluated by amplifying serial dilutions of the cDNA template. For cross-sample comparisons of results obtained using various treatments, cytokine mRNA levels were first normalized to *β*-actin mRNA levels. Results are presented as the mean and SD of three independent experiments.

### 2.9. Statistical Analysis

ANOVA and post hoc tests were performed using the ystat2004.xls statistical software package (Igakutosho Shuppan, Tokyo, Japan). One-way ANOVA with the post hoc Dunnett test or Student-Newman-Keuls test was used to determine the significance of differences in DPP-IV inhibitory assay and immunoreactivity assay, respectively. Differences were considered significant at *P* < 0.05.

## 3. Results and Discussion

### 3.1. Construction of the rBLG-Secreting NZ9000 Strain

Genetically engineered LAB have recently emerged as suitable vehicles for the delivery of therapeutic proteins (e.g., antigens of pathogenic bacteria, allergen, and immunomodulators such as cytokines) to the intestinal mucosa [17]. Many heterologous gene expression systems for LAB have been developed [18–20]. The nisin-controlled gene expression (NICE) system, which utilizes a nisin-inducible promoter to express downstream genes [21], is one of the most efficient expression systems and is commonly used with* L. lactis* and other LAB [22, 23].

**Figure 2:**
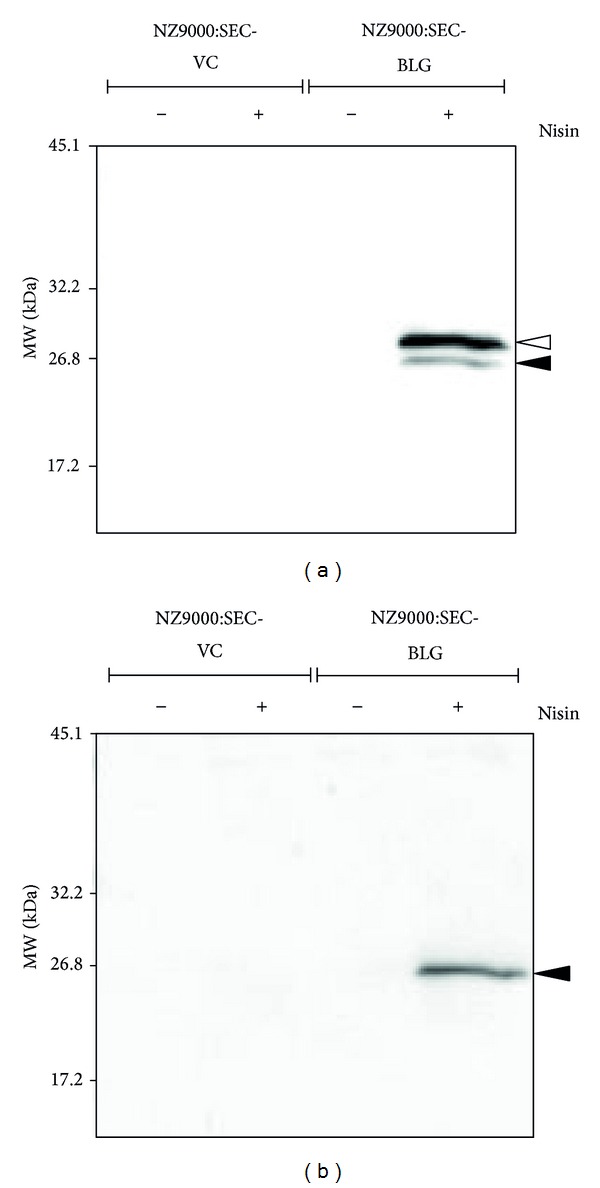
Secretion of rBLG by NZ9000. Two rNZ9000 strains were cultured with (+) or without (−) 10 ng/mL of nisin. Protein extracts from cells (a) or culture supernatants (b) were analyzed by Western blotting with a 6x His-tag Ab.* White* and* black* arrowheads indicate the secretory rBLG precursor (pre-rBLG, 27 kDa) and the secretory form of rBLG (24.3 kDa), respectively.

In the current study, we constructed the secretion plasmid pNZ8148#2:SEC (Figure 1(a)), which is a modified form of pNZ8148, the standard plasmid used in the NICE system. The pNZ8148#2:SEC plasmid contains sequences encoding the nisin-inducible promoter (*P*_nis_),* usp45* signal peptide (SP_*usp*45_), 6x His-tag, a Factor Xa recognition site, and a terminator. This plasmid was cloned from the unmutated BLG gene (Figure 1(b)) and then introduced into NZ9000, resulting in the generation of NZ9000:SEC-BLG. The NZ9000:SEC-BLG strain was induced with nisin, and rBLG expression and secretion were validated by Western blotting with an anti-6x His-tag Ab. A strong secretory rBLG precursor signal (pre-rBLG, 27 kDa) and a weak band representing the secretory form of rBLG (24.3 kDa) were observed on Western blotting of the cell extract of nisin-induced NZ9000:SEC-BLG (Figure 2(a)). Moreover, a single band corresponding to rBLG was detected in the culture supernatant of nisin-induced NZ9000:SEC-BLG (Figure 2(b)). No signal was detected in analyses of protein extracts of the nisin-induced NZ9000:SEC-VC and noninduced NZ9000 strains (Figure 2). We clearly demonstrated that NZ9000:SEC-BLG intracellular expression of pre-rBLG is dependent on nisin stimulation and that this protein is subsequently secreted by the cell's secretory machinery [18].

### 3.2. Optimal Conditions for rBLG Secretion by NZ9000

**Figure 3:**
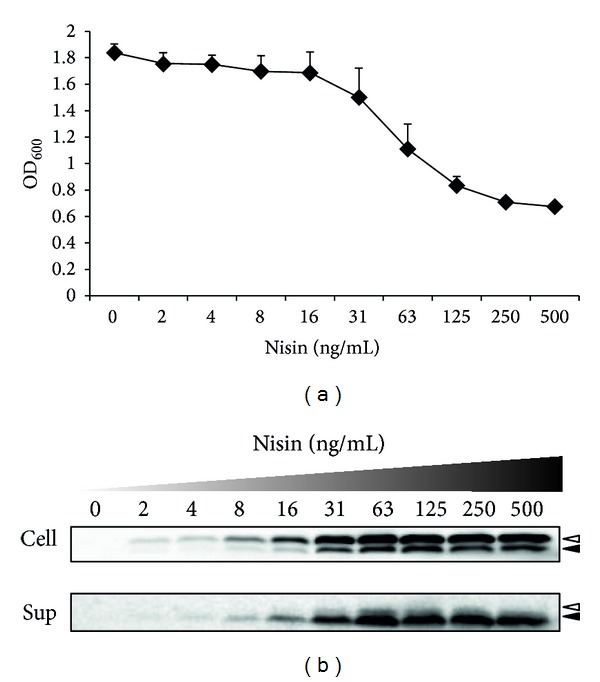
Determination of the optimal nisin concentration for secretion of rBLG by NZ9000. (a) Strain NZ9000:SEC-BLG was cultured with various concentrations of nisin (0 to 500 ng/mL). After 3 h of incubation, the OD_600_ of each sample was measured. The mean and SD from three independent experiments are shown. (b) Protein extracts from cells (CELL, above) or culture supernatants (SUP, below) were analyzed by Western blotting with a 6x His-tag Ab. Representative images from three independent experiments are shown.* White* and* black* arrowheads indicate the secretory rBLG precursor (pre-rBLG, 27 kDa) and the secretory form of rBLG (24.3 kDa), respectively.

Previous studies suggested that the amount of a protein expressed in an engineered LAB may be increased in a nisin-concentration-dependent manner using the NICE system [24]. We addressed this possibility in our previous research [13, 14]. In this study, we show that rBLG expression and secretion are nisin-concentration dependent (Figure 3(b)). Inhibition of bacterial proliferation (Figure 3(a)) and an increase in the intensity of a band corresponding to pre-rBLG in the culture supernatant (Figure 3(b)) were observed in NZ9000:SEC-BLG cultured with nisin at concentrations greater than 16 ng/mL.

Nisin, a typical type-A(I) lantibiotic produced by some* L. lactis* strains, exhibits bactericidal activity against a wide range of Gram-positive bacteria [25]. The mechanism of nisin's antimicrobial action is thought to involve (i) inhibition of cell wall biosynthesis by scavenging of the peptidoglycan precursor lipid II and (ii) lysis of the cell membrane by the formation of pores [26]. Our observations suggest that although the level of rBLG production by NZ9000:SEC-BLG increases in a nisin-concentration-dependent manner, exposure to high nisin concentrations (i.e., >16 ng/mL) results in significant cell damage. Based on these results, 15 ng/mL was determined to be the optimal nisin concentration for maximizing rBLG secretion.

### 3.3. Purification of rBLG Produced by NZ9000

**Figure 4:**
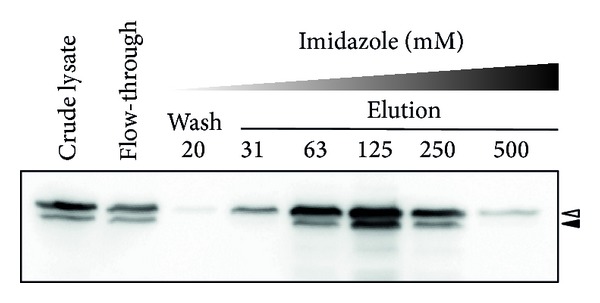
Purification of rBLG secreted by NZ9000. Expression of rBLG by NZ9000 was induced in a 4 L large-scale culture. The cell extract (*crude lysate*) was prepared as described in Section 2 and then passed through a HisTrap HP column (*flow-through*). The column was washed to remove nonadsorbed protein (*wash*) and then eluted (*elution*) with wash buffer containing 20 mM imidazole and elution buffers containing 31 to 500 mM imidazole. All fractions were analyzed by Western blotting with a 6x His-tag Ab.* White* and* black* arrowheads indicate the secretory rBLG precursor (pre-rBLG, 27 kDa) and the secretory form of rBLG (24.3 kDa), respectively.

Expression of rBLG by strain NZ9000:SEC-BLG was induced in a 4 L large-scale culture, after which the protein was isolated and purified. The cellular crude lysate was passed through a HisTrap HP column and the flow-through was collected. The column was washed to remove nonadsorbed proteins and the adsorbed proteins were eluted with various concentrations of imidazole (20 to 500 mM). To confirm the purification of rBLG, all fractions were analyzed by Western blotting with an anti-6x His-tag Ab (Figure 4). Bands corresponding to pre-rBLG and/or rBLG were observed in each of the eluted fractions. Pre-rBLG and rBLG were particularly abundant in the fractions eluted with 63 and 125 mM imidazole. However, highly purified rBLG samples (i.e., fractions eluted with 250 and 500 mM imidazole) were used in subsequent experiments.

### 3.4. Inhibition of DPP-IV Activity by Trypsin-Digested rBLG

**Figure 5:**
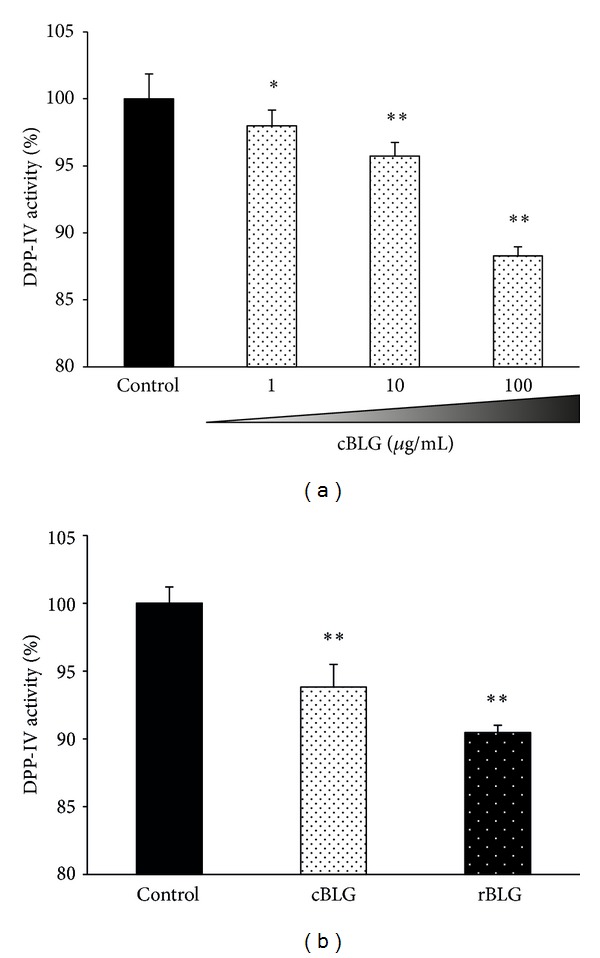
Inhibition of DPP-IV activity by trypsin-digested rBLG. Various concentrations of trypsin-digested cBLG (a) or trypsin hydrolysates of 50 *μ*g/mL of either cBLG or purified rBLG (b) were evaluated for inhibitory activity against DPP-IV using a DPP-IV Drug Discovery Kit. Concentrations indicated are the protein concentrations before trypsin treatment. Data are expressed as percent activity remaining in test samples versus that in the control (no sample added).* Black*,* dotted white*, and* dotted black *bars indicate control, cBLG, and purified rBLG (rBLG), respectively. Values represent means and error bars indicate SD (*n* = 3). *P < 0.05; **P < 0.01 versus control.

Trypsin-digested cBLG inhibited the activity of the DPP-IV enzyme in a concentration-dependent manner (Figure 5(a)). The inhibitory activity of trypsin-digested rBLG against DPP-IV was similar to that of cBLG (Figure 5(b)). Uchida et al. also showed that DPP-IV inhibitory activity of trypsin-digested BLG was exerted in a concentration-dependent fashion, and IC_50_ value was 210 *μ*M [8]. Thus, highly concentrated tryptic hydrolysate of rBLG may efficiently inhibit DPP-IV activity. Other studies have shown that some peptides produced by trypsin digestion of BLG inhibit DPP-IV activity [6, 8]. Particularly, the pentapeptide IPAVF exhibits the strongest inhibitory activity [6]. The strong inhibition of DPP-IV activity observed with trypsin-digested rBLG in this study was probably because of the involvement of these peptides within the rBLG.

### 3.5. Immunoreactivity of rBLG

Vaccines using genetically modified LAB as mucosal antigen delivery vehicles have been tested in mouse models of allergies to house dust mites [27–29], eggs [30], birch pollen [31], Japanese cedar pollen [32], and cow's milk [33]. These studies demonstrated suppression of the allergic response by LAB vaccines through induction of immune response by T-helper (Th)1 or regulatory T cells. Indeed, Adel-Patient et al. showed that oral pretreatment with a BLG-producing* L. lactis* strain prevents Th2-type immune responses through a reduction in BLG-specific IgE production and the upregulation of specific Th1-type IgG2a and fecal IgA production [34]. Therefore, we also investigated the immunoreactivity of* L. lactis*-produced rBLG using a simple* in vitro* assay system that we previously developed [13, 14]. In this study, IL-13 mRNA expression was significantly enhanced in a dose-dependent manner in splenocytes stimulated with cBLG as compared with medium-stimulated control cells (Supplemental Figure 1 available online at http://dx.doi.org/10.1155/2014/393598). Similar results were observed with cells stimulated with purified rBLG (Supplemental Figure 1). These results indicate that rBLG demonstrates immunoreactivity as strong as that of cBLG. Hence, NZ9000:SEC-BLG may be useful not only in managing T2D but also in therapies to treat or prevent allergies to cow's milk.

## 4. Conclusions

We developed a* L. lactis* strain (NZ9000 : SEC-BLG) that efficiently secretes rBLG. The rBLG produced by this strain demonstrates both strong allergenicity and inhibition of DPP-IV activity following trypsin digestion. The NZ9000:SEC-BLG strain may prove useful in therapies for treating T2D and allergies to cow's milk.

## Supplementary Material

Supplemental Figure 1: Immunoreactivity of rBLG. Splenocytes isolated from cBLG-sensi1zed BALB/c mice were stimulated in medium with or without (Med; white bar) 50 or 100 *μ*g/mL of cBLG (cBLG; dotted-white bar) or purified rBLG (rBLG; dotted-black bar). After 72 h, IL-13 mRNA levels were measured using real-time qPCR. Values represent means and error bars indicate SD (n=3). Items indicate with different letters (i.e. a, b, c, and d) were significantly different (p<0.01). Similar results were obtained from three different mice
