# Supplementary material for: Generation of Dipeptidyl Peptidase-IV-Inhibiting Peptides from β-Lactoglobulin Secreted by Lactococcus lactis
Source: Biomed Res Int. 2014 Aug 3;2014:393598. doi: 10.1155/2014/393598 (PMC4137494; doi:10.1155/2014/393598)
Supplement: Supplementary file 1 — Supplemental Figure 1: Immunoreactivity of rBLG. Splenocytes isolated from cBLG-sensi1zed BALB/c mice were stimulated in medium with or without (Med; white bar) 50 or 100 μg/mL of cBLG (cBLG; dotted-white bar) or purified rBLG (rBLG; dotted-black bar). After 72 h, IL-13 mRNA levels were measured using real-time qPCR. Values represent means and error bars indicate SD (n=3). Items indicate with different letters (i.e. a, b, c, and d) were significantly different (p<0.01). Similar results were obtained from three different mice [file 393598.f1.pdf]

Supplemental Figure 1

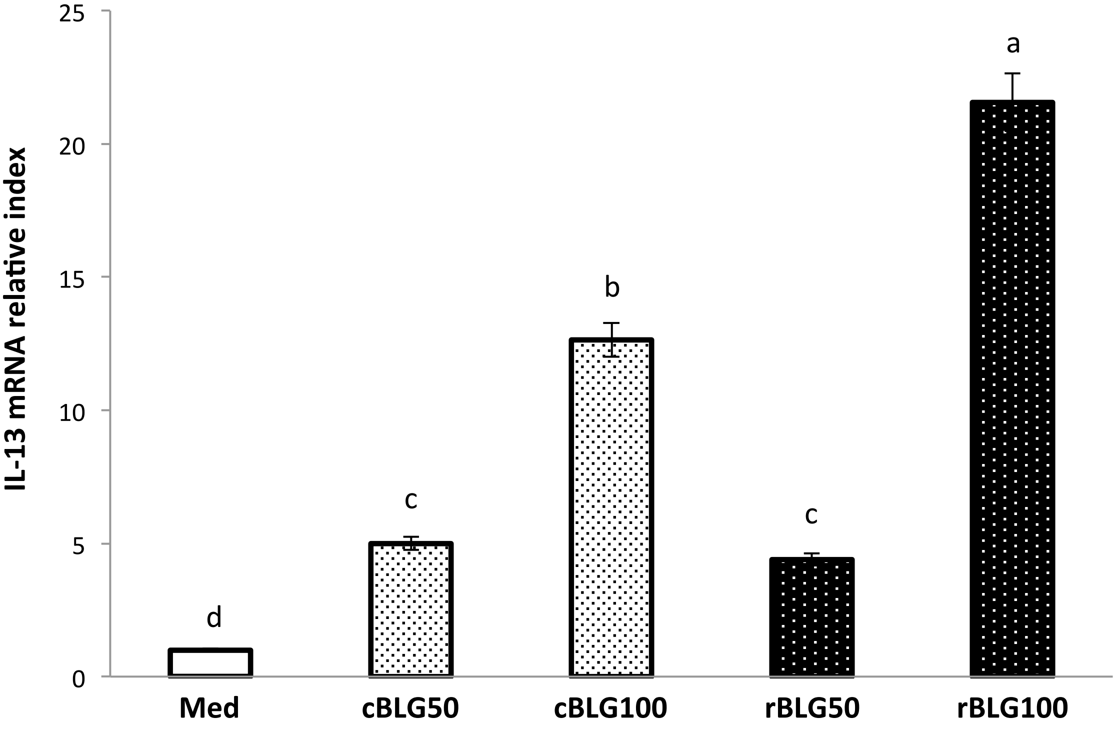

Supplemental Figure 1: Immunoreactivity of rBLG. Splenocytes isolated from cBLG-sensitized BALB/c mice were stimulated in medium with or without (*Med; white bar*) 50 or 100 µg/mL of cBLG (c*BLG; dotted-white bar*) or purified rBLG (*rBLG; dotted-black bar*). After 72 h, IL-13 mRNA levels were measured using real-time qPCR. Values represent means and error bars indicate SD (*n*=3). Items indicate with different letters (i.e. *a*, *b*, *c, and d*) were significantly different ($p<0.01$). Similar results were obtained from three different mice.
